# Spatial and Temporal Distribution of Febrile Illnesses in Uganda: Case Studies of Brucellosis, Malaria and Typhoid Fever (2016–2023)

**DOI:** 10.1155/jotm/4081643

**Published:** 2025-12-11

**Authors:** Freda Loy Aceng, Emma Sam Arinaitwe, Allan Muruta, Inaki Deza-Cruz, Abel Bulamu Ekiri

**Affiliations:** ^1^ Department of Integrated Epidemiology, Surveillance and Public Health Emergencies, Ministry of Health, Kampala, Uganda, moh.koshi.gov.np; ^2^ The Royal (Dick) School of Veterinary Studies, The University of Edinburgh, Easter Bush Veterinary Centre Roslin, Midlothian, EH25 9RG, UK, ed.ac.uk; ^3^ Department of Comparative Biomedical Sciences, School of Veterinary Medicine, Faculty of Health & Medical Sciences, University of Surrey, Guildford, UK, surrey.ac.uk

**Keywords:** brucellosis, febrile, human, illnesses, malaria, spatial, temporal, typhoid fever, Uganda

## Abstract

**Background:**

Febrile illnesses can have devastating effects on the health, wellbeing and productivity of infected individuals. Fever is one of the most frequent reasons for seeking medical care globally. In Uganda, malaria is a major cause of fever and other nonmalarial causes such as typhoid and brucellosis. The aim of this study was to improve our understanding of the spatial and temporal distribution of malaria, typhoid fever and brucellosis in Uganda in order to inform the management and control of these diseases.

**Methods:**

A retrospective analysis of data was conducted on human brucellosis, typhoid fever and malaria cases reported through the national disease surveillance system from 2016 to 2023. The data were downloaded from the health management information system into Microsoft Excel. The total number of malaria cases, typhoid fever cases and brucellosis cases over this period were 110,134,705, 1,572,162 and 361,563, respectively. Descriptive analyses were conducted using Epi Info, spatial distribution using Quantum Geographic Information System (QGIS) software. Choropleth maps were created showing cases per 100,000.

**Results:**

Nationally, from 2016 to 2023, the prevalence for malaria, typhoid fever and brucellosis varied from 43,316 to 29,271; 538 to 445 and 151 to 89 cases/100,000 population, respectively. From 2016 to 2023, there was an overall 4% decrease in the malaria prevalence/100,000, 2% decrease in the typhoid fever prevalence/100,000 and 8% decrease in the brucellosis prevalence/100,000. The burden of malaria and the nonmalarial febrile illnesses, typhoid fever and brucellosis varied across regions throughout the eight years. Overall, the northern region had the highest prevalence/100,000 for malaria and brucellosis, while the central region had the lowest prevalence for both diseases. The central region had the highest prevalence for typhoid fever.

**Conclusions:**

The study revealed the disproportionate burden of malaria and nonmalarial febrile illnesses, typhoid fever and brucellosis. The findings suggest a need to review the existing national malaria control program, to strengthen measures to mitigate the risk of typhoid fever infection and multisectoral prevention and control of brucellosis in the most affected regions and districts.

## 1. Introduction

Febrile illnesses can have devastating effects on the health, wellbeing and productivity of infected individuals. Depending on the causative agent, febrile illnesses can result in varying morbidity and mortalities. In Africa, several diseases cause acute febrile illness [1]. As such, one of the greatest challenges that clinicians face is identifying febrile patients with a potentially serious infection who need further clinical investigation and/or treatment [2]. Patients suffering from febrile illnesses often show common symptoms such as fever, headache, joint pain and back pain hence making accurate diagnosis challenging if based on only clinical presentation [3]. In circumstances where diagnostic testing availability is limited, clinicians utilise clinical syndromes to diagnose illness and guide the patient clinical management [4].

Fever is one of the most frequent reasons for seeking medical care globally [2]. In physiological terms, fever is defined as “a state of elevated core temperature, which is often, but not necessarily, part of the defensive response of multicellular organisms to invasion by live microorganisms or inanimate matter recognized as pathogenic or alien by the host” [5, 6]. Fever is measured as a rectal temperature of ≥ 38.0°C, oral temperature of ≥ 37.6°C, axillary temperature of ≥ 37.4°C and tympanic membrane of ≥ 37.6°C [7]. The affected patient may complain of fever, or of symptoms resulting from fever such as headache or general body pain [2]. The disorders that cause fever are broadly categorised as infectious, neoplastic and inflammatory [6]. Pathogenic fever is a typical characteristic of infectious disease [8].

In Africa, including Uganda, malaria is a major cause of fever. Several nonmalarial causes of fever also exist such as typhoid, and the zoonotic disease, brucellosis. Malaria is endemic in Africa and is often associated with fever [5], as such, healthcare professionals tend to screen for malaria in affected patients more frequently compared to other febrile causing illnesses [3, 9]. Malaria is a mosquito‐borne disease affecting humans caused by *Plasmodium* parasite species. It majorly spreads to people through the bites of infected female Anopheles mosquitoes [5]. Symptoms depend on the severity of the disease and include fever and flu‐like illness, chills, headache, muscle aches, tiredness, nausea, vomiting, diarrhoea, kidney failure, seizures, mental confusion and coma [10]. In 2022, there were an estimated 249 million malaria cases and 608,000 deaths globally and the highest burden was reported in Africa estimated at 233 million malaria cases and 580,000 deaths [5]. In 2021, the World Health Organisation African Region reported an estimated 234 million cases, accounting for about 95% of cases globally. Uganda (5%) Nigeria (27%), the Democratic Republic of the Congo (12%) and Mozambique (4%) accounted for almost half of all cases globally [11].

Nonmalarial febrile illnesses also occur in Africa and may be caused by viral and bacterial infections [9, 12]. In Uganda, the common causes of nonmalarial febrile illness include typhoid fever and the zoonotic infection, brucellosis [13, 14]. Although typhoid fever and brucellosis are documented in Uganda, there is limited evidence on the geographical and temporal distribution of these diseases, and the extent to which misdiagnosis of malaria and nonmalarial fever occurs. Typhoid fever is a fatal infection in humans caused by the bacterium *Salmonella typhi* and symptoms include prolonged fever, fatigue, headache, nausea, abdominal pain and constipation or diarrhoea. It is spread through contaminated water or food [15]. In 2019, an estimated 9 million typhoid cases and 110,000 deaths worldwide were reported [15]. The Global Burden of Disease study estimated 56,135 typhoid cases with 657 deaths in 2017 [16]. In sub‐Saharan Africa, there are 7.2 million cases of typhoid fever annually [17]. In Uganda, the incidence rate of typhoid fever was 160 cases per 100,000 persons per year from 2013 to 2016 [18]. A systematic review in Ethiopia found that the estimated pooled prevalence of typhoid fever based on blood and stool culture was 3%, and with Widal test was 33% [19].

Brucellosis is a zoonotic bacterial disease caused by *Brucella* species that affects humans and animals, primarily cattle, swine, sheep, goats and dogs. The disease is transmitted through direct contact with infected animals, eating or drinking contaminated animal products or inhaling airborne agents [16, 17]. It causes symptoms such as high fever, weakness, malaise, low back pain, lack of appetite, abdominal pain, vomiting and weight loss [20–22]. Over 500,000 new human cases of brucellosis are reported globally each year [23]. A systematic review and meta‐analysis conducted on studies in Ethiopia from 2015 to 2024 found that the overall pooled seroprevalence of brucellosis was 5.0%, higher in humans at 6.9% and lower in cattle at 3.5% [24]. In Uganda, the prevalence of human brucellosis in 2016 was 11% in the general population in the rural area [25].

Febrile illnesses are often unidentified and misdiagnosed in Africa including Uganda. A review of studies on the prevalence of fever of unidentified aetiology reported that most causes of fever in adolescent and adult febrile patients in East Africa remain unidentified, and there is misdiagnosis of febrile cases as malaria, which results in underestimating other causes of fever [26]. A study in Ethiopia revealed that typhoid fever, typhus, brucellosis and malaria were detected among symptomatic individuals presenting with febrile illness–related symptoms [27]. The same study also highlighted the challenge of misdiagnosing brucellosis cases as malaria or other diseases because of reliance on only clinical symptoms to differentiate the diseases causing fever [27].

Like other African countries, determining the causes of fever in Uganda remains a challenge due to several reasons including diagnostic limitations. Most cases of fever are often diagnosed and treated as malaria based on syndromes without appropriate tests being performed. Usually, it is only when the fever is unresponsive to antimalarial treatment that additional tests are performed. Lack of or delayed diagnostic testing and appropriate treatment may result in worsening of the patient condition and sometimes death. It is therefore important to identify the causative pathogen through laboratory diagnosis and provide relevant information to guide the clinical management of febrile patients.

The limited evidence on the geographical and temporal distribution of nonmalarial febrile illnesses such as brucellosis and typhoid, and the extent to which misdiagnosis of malaria and nonmalarial fever occurs remains a key knowledge gap in Uganda. The aim of this study was to improve our understanding of the spatial and temporal distribution of typhoid fever and brucellosis in addition to malaria in Uganda. Findings from this study will provide baseline data to help identify areas with high prevalence of nonmalarial febrile illness and inform future studies and interventions aimed at improving the management of nonmalarial febrile illnesses and addressing the challenges related to access to proper diagnostics of febrile illnesses.

## 2. Materials and Methods

### 2.1. Study Design

We conducted a retrospective analysis of data on human brucellosis, typhoid fever and malaria cases reported through the national disease surveillance system of the Ministry of Health, Uganda, from 2016 to 2023.

### 2.2. Study Setting

Uganda is a country in East Africa with an estimated population of 44, 212,800 [28]. It is stratified into various administrative units which include districts, counties, subcounties, parishes and villages. Currently, there are 4 regions, namely, eastern, western, central and northern, 146 districts and 10 cities [29]. Health care services are provided through a hierarchical structure of facilities including National Referral Hospitals, Regional Referral Hospitals, General hospitals and Health Centres (HC) IV, HC III and HC II [30].

### 2.3. Data Source

Retrospective data on malaria, typhoid fever and brucellosis were obtained through the national disease surveillance system for the period 2016 to 2023. The total number of malaria cases, typhoid fever cases and brucellosis cases over this period were 110,134,705, 1,572,162 and 361,563, respectively. The key inclusion criterion was use of only data collected between 2016 and 2023, and data collected outside this period were excluded. Data source completeness was shown by the availability of number of cases for the three illnesses for each of the 146 districts in Uganda. Data for each district that were considered outliers based on comparisons with the numbers across the years, were cross‐checked with the source records in the health management information system (HIMS) and corrected.

The Ministry of Health routinely monitors the disease burden in the country using the HMIS which captures data from both public and private health facilities in the country [30]. The disease surveillance reporting system follows a tiered approach from the community to the national level through the district health information system Version‐2 (DHIS2) which is a web‐based open‐source HIMS for gathering, validation, analysis and presentation of aggregate and patient‐based statistical data [31].

At the community level, the village health teams (VHTs) are trained to carry out routine surveillance, which includes identifying diseases based on clinical signs and using a standardised reporting system to report to the next hierarchical level. The community‐based disease surveillance focal persons link the patient to a nearby health facility using community case definitions for diseases and determine when to refer a person with these signs for treatment and notify the health facilities. At the community level, simplified case definitions are used to facilitate quick detection of priority diseases, events and conditions or other hazards in the community [32].

At the health facility level, information is initially collected as patient‐specific data using paper‐based Integrated Disease Surveillance and Response (IDSR) tools such as registers and summarised as immediate, weekly, monthly and quarterly HMIS reports [32]. All cases (suspected, probable and confirmed) are always recorded in the facility out‐patient department (OPD) or in‐patient department (IPD) register and reported using the HMIS forms (disease‐specific case‐based forms, HMIS 033a, HMIS 033b, HMIS 105 and HMIS 108). These reports are sent to the district health office of each of the 146 districts in the country where the biostatisticians enter the data into the electronic DHIS2. The monthly OPD report is an aggregated report for all OPD occurrences at each facility [32]. The data used in this study were obtained from the monthly aggregates of cases for each disease (malaria, typhoid fever and brucellosis) from the districts in the DHIS2.

### 2.4. Standard Case Definitions

There are standard case definitions recommended by the national technical guidelines for IDSR that are distributed to all health facilities together with the registers for recording. Health care workers use the case definitions to identify diseases, conditions or events [32].

### 2.5. Malaria Case Definitions

Uncomplicated malaria: Any person with fever or history of fever within 24 h without signs of severe disease (vital organ dysfunction) is diagnosed clinically as malaria (30). Confirmed uncomplicated malaria: Any person with fever or history of fever within 24 h and with laboratory confirmation of diagnosis by malaria blood film (microscopy) or other diagnostic test for malaria parasites (rapid diagnostic test [RDT]) (30). Unconfirmed severe malaria: Any patient hospitalised with severe febrile disease with accompanying vital organ dysfunction diagnosed clinically (30). Confirmed severe malaria: Any patient hospitalised with *P. falciparum* asexual parasitaemia as confirmed by laboratory tests with accompanying symptoms and signs of severe disease (vital organ dysfunction) diagnosed through laboratory (30).

### 2.6. Typhoid Fever Case Definitions

Suspected case: Any person with gradual onset of steadily increasing and then persistently high fever, chills, malaise, headache, sore throat, cough and, sometimes, abdominal pain and constipation or diarrhoea (30). Confirmed case: Suspected case was confirmed by isolation of *Salmonella typhi* from blood, bone marrow, bowel fluid or stool (30).

### 2.7. Brucellosis Case Definitions

Suspected case: Acute or insidious onset of fever AND ONE OR MORE of the following: night sweats, arthralgia, headache, fatigue, anorexia, myalgia, weight loss, arthritis/spondylitis, meningitis or focal organ involvement (endocarditis, orchitis/epididymitis, hepatomegaly, splenomegaly) (30). Confirmed case: A suspected case with confirmatory laboratory diagnosis by way of culture (where available) and identification of *Brucella* spp. or evidence of a fourfold or greater rise in *Brucella* antibody titre on Brucella Agglutination Test (30).

### 2.8. Data Analysis

Data on reported cases for malaria, typhoid fever and brucellosis for the period 2016 to 2023 was downloaded from the HIMS in Microsoft Excel spreadsheets and exported to Epi Info version 7.2.0 (Centres for Disease Control and Prevention, Atlanta, USA) for analysis. Descriptive analysis, including computation of annual and regional prevalence per 100,000 population from 2016 to 2023, was performed to show the temporal distribution of the febrile illnesses. The percentage changes were derived by comparing prevalence in 2023 with 2016. The spatial distribution of diseases by district and region and by year was described using Quantum Geographic Information System (QGIS) software, and choropleth maps for Uganda were created to show the cases per 100,000. Because the data were aggregated at district and regional levels, inferential statistical tests such as Chi‐square were not applied, as the primary aim was to describe rather than compare prevalence across strata.

## 3. Results

### 3.1. Prevalence of Malaria, Typhoid Fever and Brucellosis

**Figure 1:**
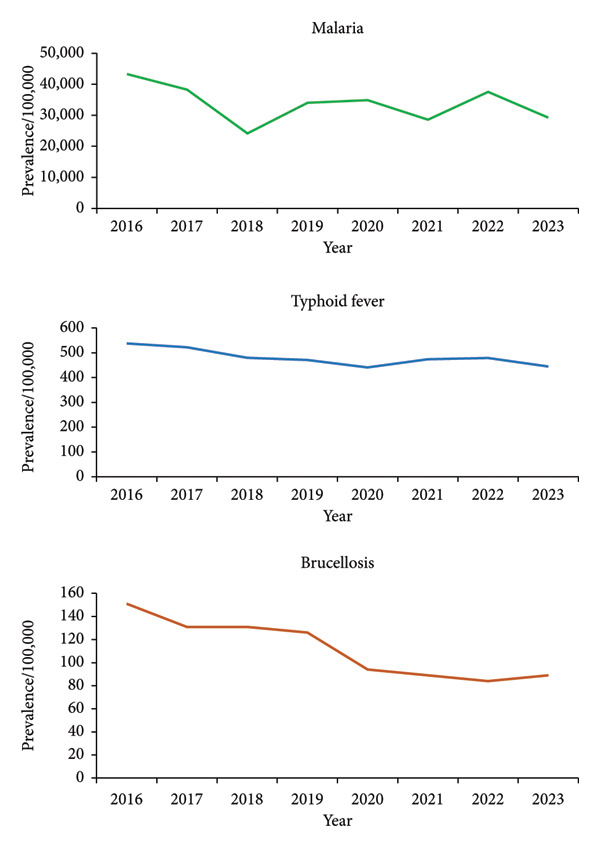
Graphs showing prevalence/100,000 population for malaria, typhoid fever and brucellosis in Uganda, 2016–2023.

Table 1 shows the prevalence/100,000 population of malaria, typhoid fever and brucellosis nationally and per region in Uganda from 2016 to 2023. The total number of malaria cases recorded were 110,134,705 (33,589/100,000 population) (Table 1). The malaria prevalence/100,000 population at the national level varied from 43,316 to 29,271 cases/100,000 population from 2016 to 2023 (Table 1, Figure 1). The total number of typhoid fever cases recorded were 1,572,162 (479/100,000 population) (Table 1). The typhoid fever prevalence/100,000 population varied from 538 to 445 cases/100,000 population from 2016 to 2023 (Table 1, Figure 1). The total number of brucellosis cases recorded was 361,563 (prevalence = 110 cases/100,000 population) (Table 1). The brucellosis prevalence/100,000 population at the national level gradually reduced from 151 to 89 cases/100,000 population from 2016 to 2023 (Table 1, Figure 1).

**Table 1:** Prevalence/100,000 population of malaria, typhoid fever and brucellosis nationally and per region in Uganda, 2016–2023.

Regions	Years								
	2016	2017	2018	2019	2020	2021	2022	2023	Total (2016–2023)
*Malaria*									
National	43,316	38,249	24,188	34,020	34,866	28,605	37,532	29,271	33,589
Northern	59,092	52,634	44,164	71,372	68,727	55,286	63,654	51,043	58,303
Eastern	46,384	42,111	26,451	30,480	33,682	33,180	45,618	34,053	36,415
Central	29,940	31,539	18,700	25,053	22,676	17,200	23,074	19,346	23,187
Western	39,575	31,262	13,737	19,545	23,019	17,781	25,236	18,916	23,341

*Typhoid fever*									
National	538	522	480	471	441	474	479	445	479
Northern	339	339	235	221	212	177	167	176	229
Eastern	363	376	295	322	299	303	319	341	287
Central	865	796	814	818	777	916	839	765	824
Western	433	460	405	380	319	336	409	337	383

*Brucellosis*									
National	151	131	131	126	94	89	84	89	110
Northern	118	147	183	143	99	110	94	96	122
Eastern	136	108	105	125	93	98	79	83	102
Central	168	129	109	118	80	70	64	70	99
Western	135	117	105	92	75	65	68	76	90

*Note:* ‘Total’ represents the mean prevalence per 100,000 population across the 8‐year period.

### 3.2. Temporal Distribution of Malaria, Typhoid Fever and Brucellosis Cases

At the national level, there was an overall 4% decrease in the malaria prevalence/100,000 from 2016 to 2023. The prevalence decreased from 2016 to 2018, increased from 2018 to 2020, decreased from 2020 to 2021, increased from 2021 to 2022 and decreased from 2022 to 2023 (Figure 1). There was an overall 2% decrease in the typhoid fever prevalence/100,000 from 2016 to 2023. The prevalence decreased from 2016 to 2020, increased from 2020 to 2022 and decreased from 2022 to 2023 (Figure 1). There was an overall 8% decrease in the brucellosis prevalence/100,000 from 2016 to 2023. The prevalence decreased from 2016 to 2017, remained almost stable between 2017 and 2019, decreased from 2019 to 2020, further decreased from 2020 to 2022 and increased in 2023.

**Figure 2:**
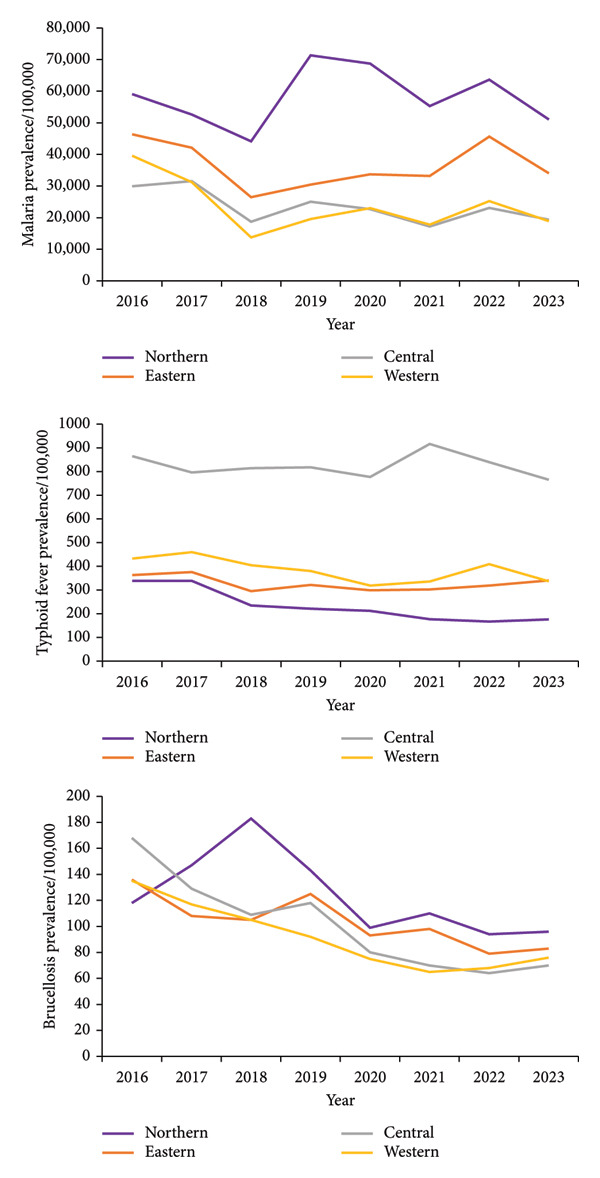
Graph showing prevalence/100,000 population for malaria, typhoid fever and brucellosis in regions of Uganda, 2016–2023.

At the regional level, the malaria, typhoid fever and brucellosis prevalence/100,000 were variable in all the four regions throughout the 10 years (Figure 2).

### 3.3. Spatial Distribution of Malaria, Typhoid Fever and Brucellosis

**Figure 3:**
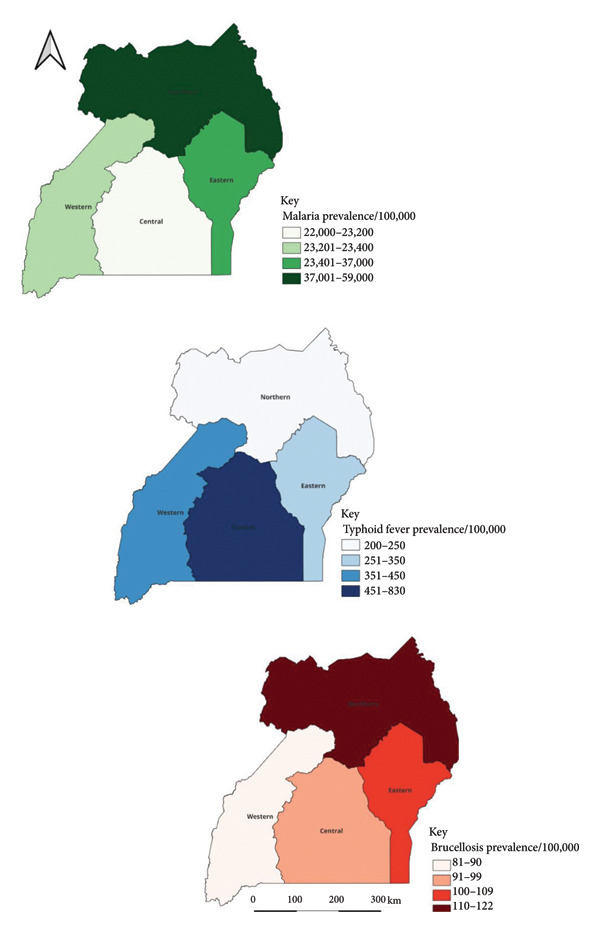
Maps showing prevalence/100,000 population of malaria, typhoid fever and brucellosis per region in Uganda, 2016–2023.

Overall and throughout the years, the northern region had the highest prevalence for malaria and brucellosis, while the central region had the lowest prevalence for both diseases (Figure 3). The central region had the highest prevalence for typhoid fever, overall and throughout the years (Figure 3).

### 3.4. Spatial Distribution of Malaria Cases

**Figure 4:**
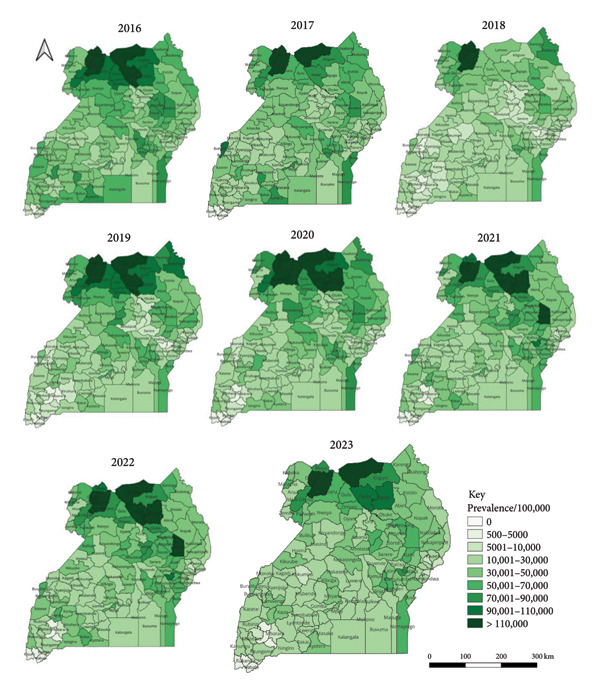
Maps showing the malaria prevalence/100,000 population in Uganda from 2016 to 2023.

At the regional level, the distribution of suspected malaria cases (prevalence/100,000 population) was highest in northern followed by eastern, western and central regions (Figure 2). At the district level (Figure 4), the distribution of suspect malaria cases (prevalence/100,000 population) was consistently high in the districts in the northern region during the 8 years including: Adjumani (155,273), Moyo (148,802) and Lamwo (144,032) in 2016; Obongi (242,022), Adjumani (156,272) and Moyo (151,809) in 2017; Obongi (264,760), Adjumani (135,762) and Moyo (124,880) in 2018; Obongi (353,948), Adjumani (174,589) and Lamwo (166,175) in 2019; Obongi (302,530), Lamwo (177,284), Adjumani (157,194) in 2020; Obongi (223,584), Adjumani (147,434), Lamwo (126,778) in 2021; Obongi (241,045), Adjumani (160,144), Lamwo (159,328) in 2022 and Obongi (205,042) and Lamwo (151,411) in 2023. The districts with the lowest prevalence of malaria were in the western region namely; Rubanda (4540) and Kisoro (5620) in 2016; Rubanda (2376) and Kisoro (3488) in 2017; Rubanda (1304) and Rukiga (1695) in 2018; Rubanda (1533), Sheema (1542) and Rukiga (1590) in 2019; Rubanda (1,402), Rukiga (1,597), Sheema (2,279) in 2020; Sheema (1,077) and Rubanda (1,153) in 2021; Rubanda (1,955), Sheema (2,616) in 2022; Rubanda (1,501) in 2023.

### 3.5. Spatial Distribution of Typhoid Fever Cases

**Figure 5:**
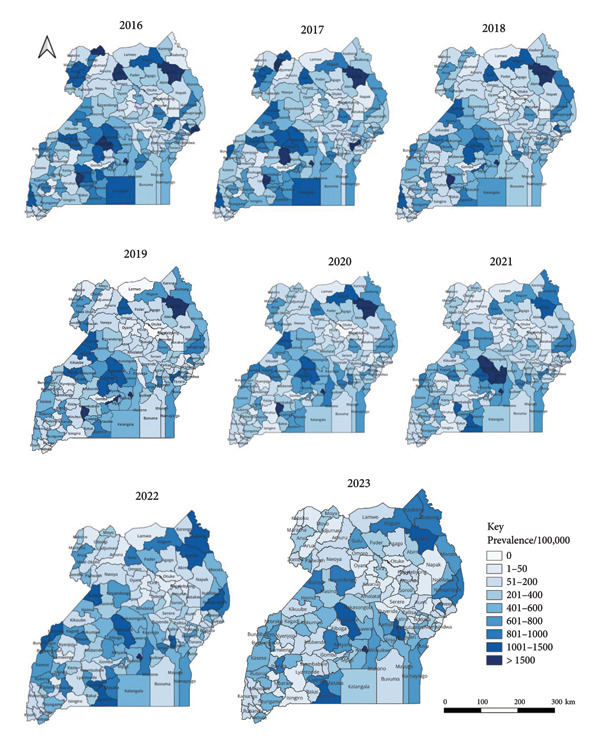
Maps showing the typhoid fever prevalence/100,000 population in Uganda from 2016 to 2023.

At the regional level, the distribution of typhoid fever cases (prevalence/100,000 population) was highest in the central region followed by western, eastern and northern regions (Figure 2). At the district level (Figure 5), the distribution of typhoid fever cases (prevalence/100,000 population) was consistently high in the districts in the central region including: Lyantonde (3377) in 2016; Lyantonde (3469) in 2017; Lyantonde (5139) in 2018; Lyantonde (4220) and Kampala (2358) in 2019; Lyantonde (2,935), Kampala (1,970), in 2020; Luwero (2,571), Kampala (2,479), Nakaseke (1,691) in 2021; Kampala (2,544), Nakaseke (1,397) in 2022 and Kampala (2,392), Kotido (1480) and Iganga (1,326) in 2023. The prevalence of typhoid fever was lowest in the following districts: Kaabong (7) and Bulambuli (9) in 2016; Kwania (6) and Rukiga (7) in 2017; Bududa (1) and Rukiga (2) in 2018; Alebtong (0) and Lamwo (0) in 2019; Alebtong (0), Otuke (2), Isingiro (2) in 2020; Adjumani (1), Kole (2), Bukedea (2) in 2021; Isingiro (2), Bukedea (9), Kapelebyong (11) in 2022; Kwania (4) and Madi‐Okollo (6) in 2023.

### 3.6. Spatial Distribution of Brucellosis Cases

**Figure 6:**
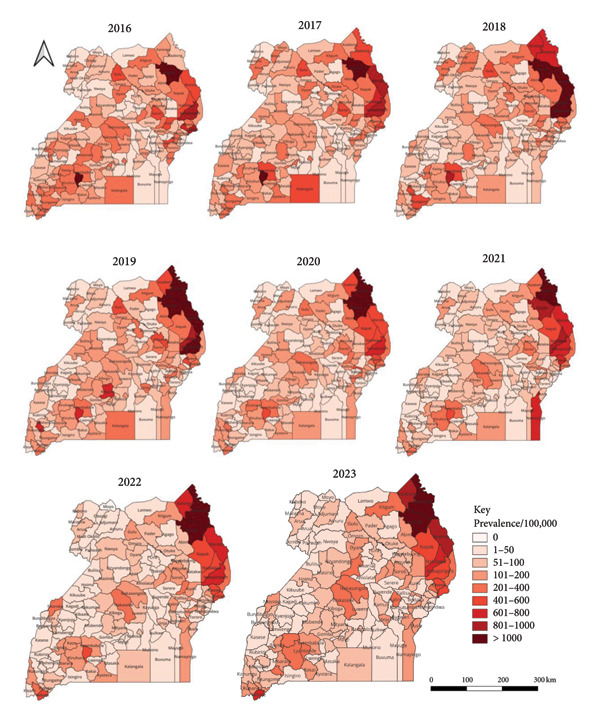
Maps showing the brucellosis prevalence/100,000 population in Uganda from 2016 to 2023.

At the regional level, the distribution of brucellosis cases (prevalence/100,000 population) was highest in northern followed by eastern, central and western regions (Figure 2). At the district level (Figure 6), the distribution of brucellosis cases (prevalence/100,000 population) was consistently high in the districts of Karamoja in the northern region during the 8 years including: Kotido (*n* = 1664) in 2016, Kotido (*n* = 1266), Lyantonde (1170), Moroto (938) and Nakapiripirit (839) in 2017; Kotido (1630), Moroto (1523), Nakapiripirit (1440), Nabilatuk (1328) and Lyantonde (925) in 2018; Kaabong (1317), Kotido (1179), Nakapiripirit (1053), Moroto (1037), Nabilatuk (977) and Lyantonde (758) in 2019; Kotido (1109), Kaabong (1007), Nakapiripirit (658) in 2020; Kaabong (1016), Nabilatuk (915), Kotido (897) in 2021; Kaabong (1482), Kotido (1122), Moroto (737) in 2022 and Kaabong (1,260), Kotido (1,137) and Moroto (899) in 2023. The prevalence of brucellosis was lowest in the districts of Yumbe (2), Buyende (3) and Bugweri (3) in 2016; Kaliro (3) and Bududa (5) in 2017; Yumbe (5) and Kaliro (6) in 2018; Pakwach (4) in 2019; Obongi (0), Buvuma (5) in 2020; Buvuma (3), Bunyangabu (4) in 2021; Moyo (0), Buvuma (1) in 2022 and Buvuma (3), Kikuube (3) in 2023.

## 4. Discussion

The study findings revealed the areas with the highest number of cases of malaria, typhoid fever and brucellosis in Uganda during the period 2016–2023. Overall, the northern region had the highest prevalence for brucellosis and malaria throughout the study period, while the central region had the lowest prevalence for both diseases. The central region had the highest prevalence for typhoid fever throughout the study period. There was an overall decrease in the prevalence/100,000 for malaria, typhoid fever and brucellosis from 2016 to 2023. The above findings provide useful baseline information that will inform interventions aimed at improving the diagnosis of febrile illnesses and subsequent treatment and patient care.

At the national level, the malaria prevalence/100,000 was 33,589. At the district level, the distribution of malaria cases (prevalence/100,000 population) was consistently high in districts in the northern region during the 8 years. The finding of high prevalence of malaria in the northern region in the current study aligns with a previous study that utilised routinely available surveillance data to analyse the spatial‐temporal patterns of malaria incidence in Uganda and showed clusters of high‐risk health facility catchments in northern and eastern regions [33]. A 2018–19 national survey of malaria in children aged 0–59 months reported prevalences of 16.9% to 9.1% based on the RDT and microscopy, respectively [34]. A study conducted among febrile under‐five refugee children attending a health centre in Mid‐Western Uganda reported malaria prevalence of 12.6% [35]. A cross‐sectional study conducted at a General Hospital in Eastern Uganda reported a prevalence of malaria of 44.4% among febrile children under 15 years of age [36].

The reasons for the differences in the regional distribution of malaria are not clear. Previous studies suggest the differences may be explained by varying factors including rainfall and humidity. A previous study in Gulu district, located in the northern region of Uganda reported that both mean monthly rainfall and relative humidity were drivers of malaria [37]. Relative humidity has been shown to play a significant role in mosquito population and malaria transmission dynamics [38]. Elsewhere, annual temperature, vegetation density, percentage of clay in soil, total amount of rainfall and distance from the nearest water body [39] have been reported to influence geographical distribution of malaria. Further investigations are required to understand the attributing factors in northern Uganda. Malaria is caused by *Plasmodium* parasite species and majorly spreads to people through the bites of infected female Anopheles mosquitoes. Other transmission methods are blood transfusion and contaminated needles. In general, risk factors for malaria in Uganda have been reported. A study in the capital city of Uganda, Kampala, identified the risk factors for malaria as history of recent overnight travel outside the city, failure to sleep under an insecticide‐treated net during travel, travel to districts which had not received indoor residual spraying (IRS), engaging in outdoor activities [40]. People at a higher risk of severe malaria infection include infants, children under 5 years, pregnant women, travellers and people with human immunodeficiency virus (HIV) or acquired immunodeficiency syndrome (AIDS) [5]. Previous studies have also reported other factors associated with malaria infection including gender, marital status, family size, altitude, house construction quality, mother’s employment status, education level, usage of long‐lasting insecticidal nets (LLINs), application of IRS, proximity to mosquito breeding sites and less robust and porous house walls [41–43].

In Uganda, the National Malaria Control Program has the mandate of providing quality assured services for malaria prevention and treatment to all people in Uganda. The program guides malaria control efforts as detailed in the Malaria Reduction Strategic Plan 2014–2020, and strategies include universal access to LLINs, parasite‐based diagnosis of malaria at all levels and strengthening malaria surveillance [44]. The use of LLINs has been demonstrated as a sustainable method for protection against malaria and has contributed greatly to reduced malaria morbidity and mortality, although the use of LLIN is still lower than the national coverage at only 69% [45]. The proportion of households with at least one LLIN was 90% in 2014; however, only 62% of households had enough LLINs to cover each household member, with the assumption that one LLIN is used by two people [34]. In 2016, about 78% of households had at least one LLIN and 62% of households had at least one LLIN for every two persons [46]. Uganda conducted another LLINs mass distribution campaign in 2017 reporting a national coverage of 97.9% [45]. Information on the extent to which the National Malaria Control Program is implemented across the various regions and related districts is scanty or lacking. Parasite‐based diagnosis of malaria at all levels requires procurement of RDTs and microscopy reagents and sundries for all health facilities. Similarly, information on the degree of access and availability of diagnostic tests and reagents at health facilities in varying regions is lacking. There is a need to address these knowledge gaps and to review the implementation of the malaria control program in the districts in the northern region where malaria was consistently high with the aim of identifying ways to decrease the prevalence of malaria in the northern region and other regions of Uganda.

Nationally, the typhoid fever prevalence/100,000 was 479 from 2016 to 2023. At the district level, the distribution of typhoid fever cases (prevalence/100,000 population) was consistently high in the districts in the central region. The findings in this study are similar to a previous study where the spatial‐temporal trends from 2012 to 2017 revealed that the majority of the disease clustering was in the central region followed by western, eastern and northern regions [47]. In the current study, the central region had the highest prevalence throughout the 8 years, and this may be attributed to the fact that the region receives the highest rainfall amounts compared to other regions which results into water stagnation and flooding with poor drainage [47]. After the heavy rains, some districts such as Nakaseke experience long droughts each year resulting in extensive water scarcity [47]. It has been observed that typhoid cases begin to rise during the rainy season in places with poor drainage and risk of flooding which provide an appropriate environment for bacterial survival and growth. Thereafter, the use of contaminated water leads to increased disease rates [47]. The lowest typhoid prevalence in the northern region may be attributed to the dryer conditions compared to other regions [47]. Other reasons for the differences in prevalence of typhoid fever at the regional level are prolonged droughts resulting into water scarcity, insufficient water resources, poor waste disposal, sanitation and hygiene, weak enforcement of the monitoring of street water quality, sale of unsafe water by vendors and improper latrine construction [47, 48]. Studies elsewhere have reported that toilet facility types, drinking water sources and population density influence geographical distribution of typhoid fever cases [49].

A lower prevalence/100,000 of typhoid fever has been reported in other studies in Uganda and the surrounding regions. In a study conducted in Uganda in 2017, the national typhoid prevalence was estimated to be 144 cases/100,000 [16]. A study conducted using retrospective data (2013–2016) in Kasese district, western region of Uganda, showed that the incidence rate of typhoid fever was 60 cases per 100,000 persons per year, and nationally, it was 160 cases per 100,000 persons per year [18]. A study conducted in East Africa including Tanzania, Kenya and Madagascar showed that the typhoid fever incidence per 100,000 people was 348 [50].

Typhoid fever is a fatal infection in humans caused by the bacterium *Salmonella typhi*, and it is spread through contaminated water or food [15]. The risk factors for typhoid fever are well documented. In 2015, a large typhoid fever outbreak in Kampala district, central region in Uganda, was caused by consumption of contaminated water from unprotected sources and drinks [51]. Elsewhere, other factors associated with typhoid fever have been identified including socioeconomic and housing transmission, foodborne transmissions, waterborne transmissions, sanitation and hygiene practices, travel‐related risk, antimicrobial agents, climate, environmental, typhoid carriers and host risk [52].

The differences in the prevalence of typhoid fever at the regional level in the current study indicate a need to further understand the explanatory factors for the regional differences and to develop and implement relevant measures to mitigate the risk of typhoid fever infection especially in the most affected districts in the central region of Uganda, such as provision of safe water and sufficient sanitation, hygiene among food handlers and vaccination. In addition, it is essential to consider the role of planning and allocation of resources and other actors in the prevention of typhoid fever.

Unlike malaria and typhoid, brucellosis is a zoonotic nonmalarial febrile illness. Nationally, the brucellosis prevalence/100,000 in the current study was 110. At the district level, the distribution of brucellosis cases (prevalence/100,000 population) was consistently high in the districts of Karamoja in the northern region during the 8 years. Brucellosis is transmitted mostly through ingestion of raw milk in the general population and other risk factors include people who work with animals or their products [20]. Brucellosis was reported in the northern region in previous studies. A previous study in northern Uganda reported a prevalence of 18.7% in febrile patients and the risk factors included rearing livestock and consumption of unpasteurised milk [53]. In another study in Karenga district, Karamoja region (part of the northern region), brucellosis was confirmed in 20% of the small ruminant herds and the close interaction of pastoralists with animals and consumption of raw milk were reported as risk factors [54]. An additional study in cattle in Karamoja region found a high brucellosis seropositivity, and this was attributed to communal land ownership and grazing [55]. The reported community practices of consuming raw milk appear to predispose the communities in the Karamoja region to high brucellosis infection as evidenced by the high prevalence in the current study.

Brucellosis has been documented in other parts of Uganda and in East Africa. A study in Kiboga district, in the central region, in an agro‐pastoral community in Uganda that consumes raw milk and milk products revealed a high human brucellosis seroprevalence at 17% [56]. Another study in Wakiso district, central region revealed an overall seroprevalence of brucellosis of 7.5% among only febrile nonmalaria out‐patients attending a health centre and raw milk consumption was associated with infection [57]. Prevalence of probable and confirmed brucellosis was 14.9% and 4.3%, respectively, among febrile patients attending a community hospital in southwestern Uganda [14]. A review of reported human brucellosis prevalence among patients attending hospitals and agro‐pastoral communities in the East African Community including Uganda, Kenya, Tanzania, Burundi, Rwanda and South Sudan ranged from 0% to 35.8% [58].

Concerted efforts from both the human and animal health sectors in the prevention and control of brucellosis are needed especially in the districts with the highest prevalence in the northern region such as in Karamoja. At the community level, there is a need to raise awareness through education of the population on risk for brucellosis transmission, food safety, occupational hygiene and laboratory safety. In animals, primarily livestock (cattle, goats and sheep), brucellosis may be prevented through serological testing and culling in areas of low prevalence. Vaccination of livestock is feasible; however, depending on the location, this approach may be hampered by high cost and limited access to vaccines. Further studies should be conducted to determine the effective intervention strategies for prevention and control of brucellosis in humans and livestock in the most affected regions and districts.

Beyond the factors discussed above, the spatial distribution of the malaria, typhoid and brucellosis may be influenced by socioeconomic status. At all levels of income, health and illness follow a social gradient whereby the lower the socioeconomic position, the worse the health [59]. Poverty is more severe in the northern region in terms of absolute numbers of poor persons and percentage of the population in poverty and the central region is the least severe [14]. The central region maintains its mean income levels well above the national average. The extent to which poverty influences the prevalence of febrile illnesses in Uganda, and the subsequent access to diagnosis and treatment is not documented. Further studies are needed to understand the potential impact of poverty level on disease prevalence in the different regions of Uganda and to inform strategies for disease control at the regional and national level.

Likewise, the distribution of health facilities may be linked to the level of febrile illness detected. The distribution of health facilities in Uganda is variable in the four regions with the central region having the highest number of total health facilities, followed by the western, eastern and lastly the northern regions [58]. In addition, the highest number of hospitals is in the central region. A study elsewhere in Ghana in 2021 reported that the poor spatial distribution of health facilities has negative implications on access to primary health care [60]. In Uganda, it is possible that the distribution of health facilities in the four regions plays a role in the level of febrile illnesses observed. Therefore, there is a need to further investigate and understand the influence of the health facility distribution and potentially other factors on the occurrence of febrile illnesses in Uganda.

At the national level, these findings emphasize the urgent need to intensify implementation of existing public health strategies for the prevention and control of febrile illnesses in Uganda. Strengthening integrated surveillance, improving access to diagnostics and promoting community‐based health education will be key to reducing disease burden. Furthermore, the observed trends align with patterns reported across East Africa, underscoring the importance of regional collaboration to harmonise surveillance systems and coordinate interventions for malaria, typhoid fever and brucellosis.

The current study has strengths and limitations. Although considered a small scope retrospective study, a key strength of this study was the provision of baseline data of the temporal and spatial distribution of malaria, typhoid fever and brucellosis in Uganda. These data can be used to inform design of interventions aimed at improving the diagnosis of these febrile illnesses and patient care and management. A limitation of the study was the use of secondary retrospective data, which may contain inconsistencies in recording and reporting, potentially introducing misclassification and reporting bias. Another potential limitation is under‐ or over‐reporting. However, where available, triangulation was used by comparing the data across multiple sources. Additionally, because the data were aggregated, it was not possible to control for potential confounders such as age, sex and socioeconomic status, or to assess individual‐level risk factors. Nonetheless, efforts were made to minimise data errors through cross‐verification with HMIS source records and by excluding outliers identified through temporal comparisons.

## 5. Conclusions

This study revealed that there was a heterogeneous spatial distribution of brucellosis, malaria and typhoid fever from 2016 to 2023. The northern region had the highest malaria and brucellosis prevalence/100,000, and the central region had the highest typhoid fever prevalence/100,000 from 2016 to 2023. It also showed an overall decrease in prevalence/100,000 population in all three diseases from 2016 to 2023.

A few important knowledge gaps were highlighted in this study. Regarding the interventions against malaria, typhoid and brucellosis, there is a need to evaluate the effectiveness of the existing interventions to prevent and control these diseases, for example, for brucellosis, evaluating interventions focussing on raising awareness through education of the population on risk factors for brucellosis transmission, food safety, laboratory safety and occupational hygiene for individuals working with livestock, and potentially vaccination of livestock. Secondly, there is a need to assess and understand the challenges faced in the most affected regions with respect to diagnostics to enable early detection of malaria, typhoid and brucellosis cases, and treatment and patient care, with a view of informing interventions that control and prevent these febrile illnesses in Uganda. In addition, to facilitate understanding of temporal distribution of these diseases, there is need for continued improvement in the local disease surveillance systems informed by existing challenges and gaps. Finally, there is need for prospective studies to estimate the human disease burden attributed to especially the nonmalarial febrile illnesses, brucellosis and typhoid, in the most impacted regions identified in the current study.

Future research should focus on developing and evaluating targeted intervention strategies tailored to high‐burden regions, such as integrated vector control programs, improved water and sanitation infrastructure and community‐based health education initiatives. In addition, advancements in diagnostic methodologies particularly point‐of‐care and multiplex testing technologies should be explored to enhance early detection and accurate differentiation of febrile illnesses. These efforts will be essential for strengthening Uganda’s surveillance capacity and guiding evidence‐based disease control programs.

We therefore call upon the Ministry of Health, regional health authorities, and development partners to use these findings to inform policy decisions, allocate resources strategically and strengthen public health interventions in the most affected regions. Future research should build on this baseline to evaluate the effectiveness of implemented strategies and advance evidence‐based approaches for the control and prevention of febrile illnesses in Uganda.

## Ethics Statement

We sought permission to use the routinely generated program surveillance data from the Uganda Ministry of Health. The study used pre‐existing routinely collected surveillance data, which are aggregated, and no patient details were provided; therefore, no ethical clearance was obtained.

## Conflicts of Interest

The authors declare no conflicts of interest.

## Funding

The authors received no funding for this work.

## Data Availability

The data that support the findings of this study are available from the corresponding author upon reasonable request.
